# Assessment of Antimicrobial Resistance and Virulence of Biofilm-Forming Uropathogenic *Escherichia coli* from Rio de Janeiro

**DOI:** 10.3390/antibiotics14090869

**Published:** 2025-08-29

**Authors:** Maria Clara F. Oliveira, Anna Luiza B. Canellas, Lidiane C. Berbert, Alexander M. Cardoso, Vitoria A. Silva, Samantha S. T. Garutti, Débora Hosana F. Rangel, Rubens Clayton S. Dias, Jamila Alessandra Perini, Claudia R. V. M. Souza, Thiago P. G. Chagas, Marinella S. Laport, Flávia Lúcia P. C. Pellegrino

**Affiliations:** 1 Laboratórios Integrados de Pesquisa em Bactérias Resistentes aos Antimicrobianos e em Desenvolvimento Galênico-LIPE, Faculdade de Ciências Biológicas e Saúde (FCBS), Universidade do Estado do Rio de Janeiro (UERJ), Rio de Janeiro 23070-200, RJ, Brazil; mariaclara.mcf68@gmail.com (M.C.F.O.); vitoriaallvesilva@gmail.com (V.A.S.); hosana503@gmail.com (D.H.F.R.); 2 Instituto de Microbiologia Paulo de Góes, Universidade Federal do Rio de Janeiro, Av. Carlos Chagas Filho, 373, Cidade Universitária 21941-902, RJ, Brazil; annaluiza@micro.ufrj.br (A.L.B.C.); samanthatufic@gmail.com (S.S.T.G.); marinella@micro.ufrj.br (M.S.L.); 3 Laboratório de Biotecnologia Ambiental, Faculdade de Ciências Biológicas e Saúde (FCBS), Universidade do Estado do Rio de Janeiro (UERJ), Rio de Janeiro 23070-200, RJ, Brazil; lidy_berbert@hotmail.com (L.C.B.); amcardosopf@yahoo.com.br (A.M.C.); 4 Instituto Biomédico, Universidade Federal do Estado do Rio de Janeiro (UNIRIO), Rio de Janeiro 22290-240, RJ, Brazil; rubens.dias@unirio.br; 5 Laboratório de Pesquisa de Ciências Farmacêuticas-LAPESF, Faculdade de Ciências Biológicas e Saúde (FCBS), Universidade do Estado do Rio de Janeiro (UERJ), Rio de Janeiro 23070-200, RJ, Brazil; jamilaperini@yahoo.com.br; 6 Laboratório de Epidemiologia Molecular e Biotecnologia, Departamento de Patologia, Universidade Federal Fluminense (UFF), Niterói 24030-215, RJ, Brazil; claudia_souza@id.uff.br (C.R.V.M.S.); tpgchagas@id.uff.br (T.P.G.C.)

**Keywords:** uropathogenic *Escherichia coli*, biofilm, antimicrobial resistance, virulence, urinary tract infection

## Abstract

**Background/Objectives:** Uropathogenic *Escherichia coli* (UPEC) is the leading cause of urinary tract infections in both community and hospital settings worldwide. Antimicrobial-resistant UPEC strains pose a significant challenge for effective antibiotic therapy. In this study, 50 bacterial isolates recovered from urine samples of patients attended in different sectors of a public hospital in Rio de Janeiro over five months were analyzed to assess antimicrobial resistance and virulence profiles through broad gene screening. **Methods:** Biofilm production was assessed using a semi-quantitative adherence assay. PCR was employed to investigate 27 resistance genes, 6 virulence genes, sequence types (STs), and phylogroups. Susceptibility to 25 antimicrobial agents was determined by disk diffusion testing. Furthermore, the pathogenic potential was evaluated *in vivo* using the *Tenebrio molitor* larvae infection model. **Results:** Most UPEC isolates were moderate or strong biofilm producers (41/50; 82%). The *sul1* and *sul2* resistance genes were the most frequently detected (58%). Two virulence gene patterns were identified: *fyuA*, *iutA*, *fimH*, *cnf1* and *fyuA*, *iutA*, *fimH* (13 isolates; 26%). ST131 and ST73 were the most common sequence types (16% each), and phylogroup B2 was the most prevalent (50%). Thirty isolates (60%) were multidrug-resistant, most of which belonged to phylogroup B2. UPEC exhibited dose-dependent lethality, causing 100% mortality at 2.6 × 10^8^ CFU/mL within 24 h. **Conclusions:** These findings reinforce the urgent need for surveillance strategies and effective antimicrobial stewardship in clinical practice.

## 1. Introduction

Urinary tract infections (UTIs) are among the most prevalent bacterial infections worldwide, accounting for approximately 400 million cases annually [1]. UTIs encompass infections of the urethra, bladder, and kidneys, presenting with heterogeneous clinical manifestations that may be classified as uncomplicated or complicated depending on the risk of progression from mild to severe disease [2]. *Escherichia coli* is the leading uropathogen responsible for both community-acquired and healthcare-associated UTIs, especially catheter-associated infections [2,3].

Uropathogenic *E. coli* (UPEC), a highly adapted colonizer of the urinary tract (UT), is the predominant etiologic agent in UTIs [1,2,3]. UPEC pathogenesis relies on an arsenal of virulence factors, including adhesins, biofilm formation, toxins, and siderophores, which collectively facilitate bacterial survival, persistence, and infection establishment within the host UT [4,5]. Adhesins, such as FimH, mediate bacterial attachment to host cells, promoting colonization and invasion as well as biofilm development [4]. Biofilms enhance bacterial persistence by protecting against host immune defenses, antimicrobial agents, and mechanical clearance by urine flow. Toxins such as hemolysin and cytotoxic necrotizing factor 1 (CNF1) contribute to tissue damage and immune evasion, while siderophores enable iron acquisition in the nutrient-limited UT environment, further supporting UPEC survival [2]. These virulent determinants underline UPEC’s success as a recurrent UTI pathogen.

UTIs represent a significant burden on healthcare systems, being a leading cause of morbidity and outpatient medical visits globally [4,6,7]. Consequently, UTIs are among the primary indications for antimicrobial prescriptions worldwide, imposing considerable socioeconomic and individual impacts [8]. First-line antibiotic therapies for uncomplicated cystitis typically include nitrofurantoin, trimethoprim-sulfamethoxazole, and fosfomycin, with nitrofurantoin and trimethoprim-sulfamethoxazole maintaining efficacy even against some extended-spectrum beta-lactamase (ESBL)-producing UPEC strains [5]. Fluoroquinolones and beta-lactams are often reserved as second-line agents [6]. However, escalating antimicrobial resistance among UPEC strains poses a serious threat to effective UTI management. In 2019, *E. coli* ranked among the top six bacterial pathogens causing antimicrobial resistance-related deaths and remains a critical priority pathogen according to the 2024 World Health Organization [7,8]. Extensive antimicrobial use for UTIs has driven the emergence and dissemination of multidrug-resistant UPEC strains, complicating therapeutic options and clinical outcomes [9].

The present study aimed to characterize outpatient and hospital UPEC isolates from Rio de Janeiro with respect to pathogenicity, virulence factors, epidemiology, and antimicrobial resistance profiles. Through comprehensive screening of resistance determinants, virulence genes, and phylogenetic groups, our results delineate current trends in UPEC antimicrobial resistance in this region, underscoring the urgent need for improved antimicrobial stewardship in Brazil.

## 2. Results

### 2.1. Bacterial Identification, Antimicrobial Susceptibility, and Biofilm Production

All 50 bacterial isolates initially identified as *Escherichia coli* by PHOENIX™ BD M150 were confirmed as *E. coli* by MALDI-TOF MS, with high confidence scores (>2.0). Biofilm production was observed in all isolates, with 15 classified as strong producers (30%), 26 as moderate (52%), and 9 as weak producers (18%). The resistance rates among the 50 UPEC isolates were as follows: β-lactam agents (72%), quinolones (56%), tetracyclines (54%), trimethoprim-sulfamethoxazole (46%), aminoglycosides (30%), fosfomycin (14%), and nitrofurans (12%). Thirty isolates (60%) were classified as multidrug-resistant (MDR), while only seven isolates (14%) were fully susceptible to all antimicrobials tested. No resistance to carbapenems was detected by disk diffusion testing.

### 2.2. Resistance and Virulence Genes

**Figure 1:**
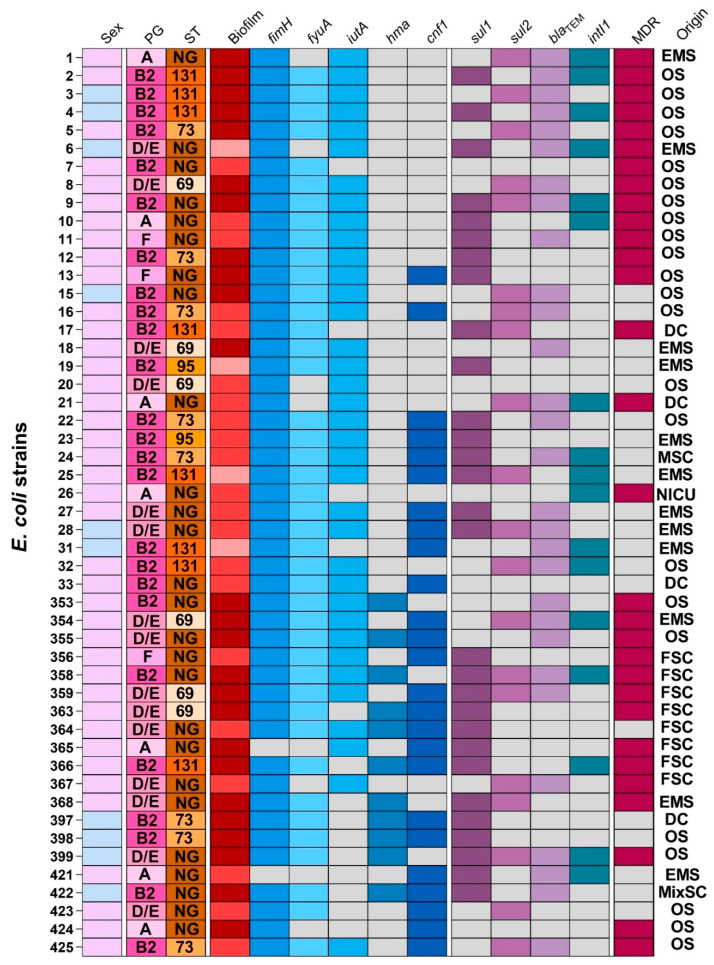
The heatmap illustrates the distribution of key virulence genes (blue gradient) and antimicrobial resistance genes (purple gradient) among 50 UPEC isolates. Biofilm formation is represented in a red gradient, ranging from light red (weak producers) to dark red (strong producers). Gray squares indicate gene absence. Patient sex is denoted by light pink (female) and light blue (male). PG: phylogenetic group; ST: sequence type; NG: non-grouped; MDR: multidrug-resistant isolate; EMS: emergency service; OS: outpatient service; DC: dialysis center; MSC: male surgical clinic; NICU: neonatal intensive care unit; FSC: female surgical clinic; MixSC: mixed surgical clinic.

A comprehensive genomic and phenotypic characterization of 50 uropathogenic *Escherichia coli* (UPEC) isolates revealed a heterogeneous distribution of antimicrobial resistance genes and virulence traits (Figure 1). Sulfonamide resistance genes *sul1* and *sul2* were detected in 58% and 40% of isolates, respectively, with co-occurrence observed in 18% of multidrug-resistant (MDR) strains. No isolates were positive for the *sul3* gene. The *bla_TEM_* gene was identified in 50% of isolates, often in conjunction with the class 1 integron integrase gene (*intI1*), which was found in 34% of strains. Beta-lactamase-encoding genes *bla_CTX_*_-*M*-*1*,*2*_, *bla_SHV_*, and *bla_GES_* were present in 24%, 12%, and 2% of isolates, respectively. The presence of these resistance determinants was strongly associated with the MDR phenotype, observed in 60% of the cohort. In terms of additional resistance genes, *aac*(6′)-*Ib*, conferring resistance to aminoglycosides, was found in 12% of strains, and *qnrS*, associated with quinolone resistance, in 2%. No isolates were positive for *bla_CTX_*_-*M*-*8*_, *bla_CTX_*_-*M*-*14*_, *bla_KPC_*, *ant*(2″)-*I*, *aac*(6′)-*Ie*-*aph*(2″)-*Ia*, *qnrA*, *qnrD*, or *mcr* gene variants associated with colistin resistance. MDR isolates were significantly more likely to harbor *sul1* (*p* = 0.004), *bla_TEM_* (*p* = 0.01), and *intI1* (*p* = 0.03).

Among the virulence factors analyzed, *fimH* was the most frequently detected gene, present in 96% of isolates. Its presence was significantly associated with strong biofilm production (Fisher’s exact test, *p* = 0.002). Siderophore-associated genes *fyuA* and *iutA* were found in 84% and 56% of strains, respectively. The cytotoxic gene *cnf1* was identified in 24% of isolates, while *hma* was less common (18%). The *ireA* gene was not detected in any isolate. Two major virulence gene patterns were identified: *fyuA*-*iutA*-*fimH*-*cnf1* and *fyuA*-*iutA*-*fimH*, both observed in 13 (26%) of the 50 UPEC isolates. A significant association was found between the multidrug resistance (MDR) status and the presence of the virulence gene *fyuA* (*p* = 0.018). Additionally, a strong correlation was observed between the level of biofilm production and the presence of the *iutA* gene (*p* = 0.004).

Clinical origin analysis indicated that the majority of MDR isolates originated from the emergency service (EMS) and outpatient sectors (OS). Notably, all isolates from the female surgical clinic (FSC) were strong biofilm producers and carried multiple virulence and resistance genes, suggesting higher risk of persistent infections (Figure 1).

### 2.3. Sequence Types and Phylogenetic Groups

Clonal analysis revealed that ST131 and ST73 were the most prevalent sequence types, each accounting for 16% of the isolates, and were predominantly associated with the B2 phylogenetic group (50%). ST131/B2 isolates consistently harbored multiple virulence and antimicrobial resistance genes, underscoring their classification as high-risk clones. In contrast, non-B2 phylogroups, including A and F, exhibited reduced virulence gene carriage and lower rates of multidrug resistance (MDR). Phylogroup B2 was significantly overrepresented among MDR isolates (Chi-square test, *p* = 0.006) and among those carrying three or more virulence genes (*p* = 0.01). Notably, ST131 isolates were almost exclusively MDR and belonged to phylogroup B2, with 87.5% (7 out of 8) exhibiting resistance to both fluoroquinolones and sulfonamides.

### 2.4. Larvae Infection

**Figure 2:**
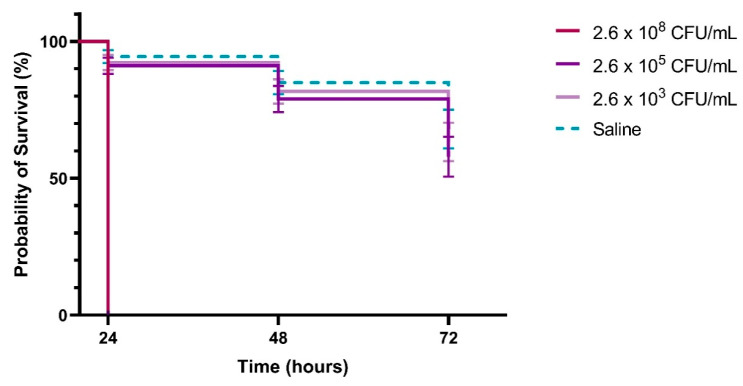
Survival curves of *Tenebrio molitor* larvae infected with the UPEC 355 isolate. Groups of 15 larvae were inoculated with varying bacterial doses and monitored every 24 h for up to 72 h post-infection. Larvae injected with sterile saline (blue dashed lines) served as negative controls. Error bars represent standard deviations at each time point.

The *T. molitor* infection model demonstrated a clear dose-dependent virulence of the UPEC 355 strain, with higher bacterial inocula resulting in markedly reduced larval survival. At the highest concentration tested (2.6 × 10^8^ CFU/mL), larval survival dropped to 0% within 24 h (Figure 2). Conversely, at the lowest inoculum (2.6 × 10^3^ CFU/mL), over 70% of larvae remained alive at the same time point.

## 3. Discussion

Antimicrobial resistance (AMR) has become a serious and urgent global public health problem and a critical threat to modern healthcare. *Escherichia coli* has been ranked among the six leading pathogens responsible for antimicrobial resistance-associated deaths in 2019 (*Staphylococcus aureus*, *Escherichia coli*, *Klebsiella pneumoniae*, *Streptococcus pneumoniae*, *Pseudomonas aeruginosa*, and *Acinetobacter baumannii*), accounting for a significant disease burden, with an estimated 929,000 deaths and over 3.5 million linked cases globally. In recognition of its clinical and public health significance, *E. coli* is listed as a critical priority pathogen by the World Health Organization (WHO) [10,11].

Uropathogenic *E. coli* (UPEC) is a subgroup of *E. coli* pathogenic strains that cause extraintestinal infections (ExPEC), specialized in causing urinary tract infections by a combination of unusual virulence characteristics, metabolic flexibility, and antibiotic resistance, making it a clinically relevant pathogen [1,2,3]. In the present study, molecular screening of resistance genes in UPEC isolates revealed a high prevalence of sulfonamide resistance genes: *sul1* (58%) and *sul2* (40%), with co-occurrence in 18% of isolates. Resistance to sulfonamides in *E. coli* is typically linked to integron-associated genes such as *sul1*, *sul2*, and *sul3*, enabling their horizontal transfer [12]. While *sul1* is part of the conserved integron 1 (*Int1*), its presence is not obligatory due to known deletions in *intI1* variants [10,13]. Conversely, *sul2* is not integron-associated, and *sul3* is rarely encountered and sometimes found in association with *intI1* [4,14]. In our study, *sul3* and integron/integrase genes (*intI1*, *intI2*, *intI3*) were absent in several *sul1/sul2*-positive isolates, potentially explaining why some strains (UPEC 19, 364, 423) did not exhibit resistance to trimethoprim-sulfamethoxazole in phenotypic assays. These findings align with a Swedish study in which 58 sulfonamide-resistant *E. coli* isolates were characterized: 11 harbored *sul1*, *20 sul2*, and 25 both genes; two *sul3*-positive isolates carried distinct plasmids and were highly resistant to sulfonamides [15]. In addition, an Iraqi study reported 100% of sulfamethoxazole-trimethoprim (SXT)-resistant *E. coli* strains carried *sul2*, and 92.9% carried *sul1*, with no detection of *sul3* [16].

Genotypic and phenotypic resistance profiles in our study were broadly concordant. Most UPEC isolates carrying *bla_TEM_*, *sul*, *ctx*, or *qnr* genes also exhibited resistance to ampicillin, sulfonamides, cephalosporins, and fluoroquinolones, respectively, and many were classified as MDR. Notably, UPEC 17 exhibited resistance to 18 of the 25 antimicrobials tested and harbored *bla_CTX_*_-*M*-*1/2*_, *aac*(6′)-*Ib*, *sul1*, and *sul2*. These results corroborate previous reports describing high *sul1*, *bla_TEM_*, and *bla_SHV_* carriage among MDR UPEC isolates [7,8,9,10,11,12,13,14,15,16,17,18,19]. All isolates in this study produced biofilm in vitro, and the fimbrial adhesin gene *fimH* was detected in 96% of isolates. Strong biofilm producers consistently harbored *fimH*, while the two *fimH*-negative isolates (UPEC 365 and 421) produced only weak or moderate biofilms. Biofilm formation facilitates bacterial persistence in the urinary tract by enhancing adhesion, protecting against host immune responses and antimicrobial agents, and allowing evasion from urine flow clearance [14,20].

Two dominant virulence gene patterns, *fyuA*, *iutA*, *fimH*, *cnf1* and *fyuA*-*iutA*-*fimH*, were found in 26% of isolates. High prevalence of *fimH*, *fyuA*, and *iutA* has previously been observed in both symptomatic and asymptomatic UTI patients [21]. The *cnf1* gene, more often associated with prostatitis, was found in 24% of isolates [22]. These virulence factors contribute synergistically to colonization, immune evasion, tissue damage, and nutrient acquisition in the iron-limited urinary tract environment [4,23,24,25,26]. Furthermore, *in vivo* validation using the *Tenebrio molitor* model demonstrated dose-dependent lethality of the UPEC 355 strain, corroborating its strong virulence phenotype. Although *T. molitor* is less commonly used than *Galleria mellonella*, both invertebrate models have proven effective in identifying high-virulence UPEC lineages [27].

Clonal analysis revealed that ST131 and ST73 were the most frequent sequence types (16% each), with ST131 notably associated with MDR and fluoroquinolone resistance. ST131 isolates also consistently carried *fimH*, in line with the global expansion of the *fimH30* sublineage [28,29,30,31]. The predominance of phylogroup B2 (50%), especially among ST131 isolates, reflects prior reports of its association with MDR and enhanced virulence [24,25]. Within phylogroup B2, 84% of isolates carried at least one *sul* gene, 64% carried *bla_TEM_*, and 44% were MDR. In contrast, non-B2 phylogroups such as A and F showed lower virulence and resistance gene carriage. Weak biofilm producers carried fewer virulence genes (1–3), while moderate and strong biofilm producers typically harbored 3–5 virulence genes (74%). These observations confirm previous reports of phylogroup B2 being enriched in high-risk, MDR UPEC clones [32]. Among strong biofilm producers, 87% (13/15) carried at least one *sul* variant, 47% carried *bla_TEM_*, and 60% were classified as MDR. These traits were significantly associated with virulence gene presence and biofilm production, indicating a convergence of resistance and pathogenicity traits.

The integrative genotypic–phenotypic profiling performed here reveals a local UPEC population dominated by high-risk clones such as ST131/B2. The near-universal detection of *fimH* gene emphasizes its importance in bladder colonization, while co-occurrence of resistance genes (*bla_TEM_*, *sul1/2*) and virulence factors (*fyuA*, *iutA*, *cnf1*) suggests coordinated acquisition, likely via mobile genetic elements including integrons. These findings echo global trends of increasing convergence between virulence and resistance, with direct implications for treatment failure and recurrent infections. The enrichment of high-risk clones in hospital sectors such as FSC (female surgical clinic) and EMS (emergency service) raises concerns about their potential role as reservoirs for nosocomial and community dissemination. Although the *T. molitor* model was applied to only one strain, its results aligned with the isolate’s resistance and virulent profiles. Further studies employing expanded *in vivo* models encompassing diverse sequence types (STs) and phylogenetic groups are warranted to better elucidate the relationship between genotype, virulence, and clinical outcome.

## 4. Materials and Methods

### 4.1. Study Design, Patients, Bacterial Isolates and Ethical Approval

Fifty uropathogenic *Escherichia coli* (UPEC) isolates were included in this study. These isolates were obtained from urine samples of 50 patients, aged between 0 and 92 years, who were clinically diagnosed with urinary tract infections (UTIs) characterized by symptomatic bacteriuria due to Gram-negative bacilli. Patients were attended in various sectors (outpatient clinics and hospital wards) of a public hospital in Niterói, in the metropolitan region of Rio de Janeiro, Brazil, between May and October 2019. The sample size was defined based on the following inclusion criteria: urine culture positive for *Enterobacteriaceae* and only one isolate per patient. Quantitative urine cultures were performed in the hospital’s microbiology laboratory, and cultures were considered positive for UTI when bacterial counts reached or exceeded 10^5^ CFU/mL. Samples were inoculated onto CHROMAGAR™ Orientation medium plates (CHROMAGAR, Saint-Denis, France) for differential isolation. Initial identification of *E. coli* was performed using the automated PHOENIX BD™ M150 system (Enzipharma Diagnóstica, Rio de Janeiro, Brazil). Confirmed isolates were stored in 10% glycerol stocks at −20 °C until further use. This study was conducted as part of a larger project approved by the hospital’s Research Ethics Committee (approval number 2.920.186/CAAE: 95984018.6.0000.5243).

### 4.2. Bacterial Identification Confirmation

Species-level identification of the bacterial isolates was confirmed using matrix-assisted laser desorption/ionization time-of-flight mass spectrometry (MALDI-TOF MS) on the Microflex LT platform (Bruker Daltonics, Bremen, Germany), following standard protocols with minor modifications [9]. Each isolate was cultured overnight on Luria–Bertani (LB) agar, and a portion of the bacterial biomass was directly transferred to a polished steel MSP 96 target plate (Bruker Daltonics). Samples were overlaid with 1 μL of 70% formic acid and allowed to air-dry. Subsequently, 1 μL of α-cyano-4-hydroxycinnamic acid (HCCA) matrix solution was added to each spot. Each isolate was analyzed in duplicate. *E. coli* DH5α was included as a positive control for species identification in every run, while *E. coli* ATCC 25922 was used as a reference calibrant. Acquired spectra were matched against the Bruker Biotyper reference library using the MALDI Biotyper 7.0 software, and identifications were interpreted based on the manufacturer’s score criteria [33].

### 4.3. Evaluation of Biofilm Production

Biofilm production was assessed using a standard semi-quantitative adherence assay in 96-well polystyrene microtiter plates, as previously described [34]. Briefly, overnight cultures of *E. coli* isolates were adjusted to the 0.5 McFarland standard and inoculated into the wells. Following incubation, wells were gently washed, and adherent biofilms were stained with 0.1% crystal violet. Each UPEC isolate was evaluated in three independent experiments, each performed in triplicate, resulting in nine measurements per isolate. *Staphylococcus aureus* ATCC 6538, a known strong biofilm producer, was used as the positive control, while sterile Luria–Bertani (LB) broth served as the negative control. Optical density (OD) was measured at 570 nm using a microplate reader. Biofilm production was classified based on OD values according to established criteria [35]. The cutoff OD (ODc) was calculated as the mean OD of the negative control plus three standard deviations (ODc = mean + 3 × SD). Classification thresholds were defined as follows: OD ≤ ODc: non-biofilm producer, ODc < OD ≤ 2 × ODc: weak biofilm producer, 2 × ODc < OD ≤ 4 × ODc: moderate biofilm producer, and OD > 4 × ODc: strong biofilm producer.

### 4.4. Antimicrobial Susceptibility Testing

Antimicrobial susceptibility of the UPEC isolates was assessed using the disk diffusion (DD) method, following the Clinical and Laboratory Standards Institute (CLSI) guidelines, 2020 edition [36]. A total of 25 antimicrobial agents (Cefar Diagnóstica Ltda., São Paulo, Brazil) were tested, including representatives from multiple antibiotic classes including β-lactams: ampicillin (AMP, 10 μg), ampicillin + sulbactam (APS, 20 μg), amoxicillin + clavulanic acid (AMC, 30 μg), cefazolin (CFZ, 30 μg), cefuroxime (CRX, 30 μg), cefoxitin (CFO, 30 μg), cefotaxime (CTX, 30 μg), ceftriaxone (CRO, 30 μg), cefepime (COM, 30 μg), aztreonam (ATM, 30 μg), piperacillin + tazobactam (PIT, 110 μg); Carbapenems: imipenem (IPM, 10 μg), meropenem (MPM, 10 μg), ertapenem (ERT, 10 μg); Quinolones/Fluoroquinolones: nalidixic acid (NAL, 30 μg), ciprofloxacin (CIP, 5 μg), norfloxacin (NOR, 10 μg), levofloxacin (LVX, 5 μg); Aminoglycosides: amikacin (AMI, 30 μg), gentamicin (GEN, 10 μg), tobramycin (TOB, 10 μg); Others: tetracycline (TET, 30 μg), nitrofurantoin (NIT, 300 μg), fosfomycin (FOS, 200 μg), sulfamethoxazole + trimethoprim (SXT, 25 μg). *Escherichia coli* ATCC 25922 was used as the quality control reference strain for the DD assays. Interpretation of inhibition zones was conducted in accordance with CLSI criteria. Isolates were classified as multidrug-resistant (MDR) when they exhibited resistance to at least one antimicrobial agent in three or more different antibiotic classes, including β-lactams, quinolones, aminoglycosides, sulfonamides, or colistin.

### 4.5. Antimicrobial Resistance Genes Screening

A total of 24 antimicrobial resistance genes (ARGs) were screened in the UPEC isolates by polymerase chain reaction (PCR). The targeted genes included β-lactamase genes: *bla_CTX_*_-*M*-*1*,*2*_, *bla_CTX_*_-*M*-*8*_, *bla_CTX_*_-*M*-*14*_, *bla_KPC_*, *bla_TEM_*, *bla_GES_*, and *bla_SHV_*; quinolone resistance genes: *qnrA*, *qnrD*, and *qnrS*; aminoglycoside-modifying enzyme genes: *aac*(6′)-*Ib*, *ant*(2″)-*Ia*, and *aac*(6′)-*Ie*-*aph*(2″)-*Ia*; sulfonamide resistance genes: *sul1*, *sul2*, and *sul3*; colistin resistance genes: *mcr*-*1* through *mcr*-*5*, and *mcr*-*7* through *mcr*-*9*. In addition, three integron-integrase genes associated with mobile genetic elements were assessed: *intI1*, *intI2*, and *intI3*. These elements are known to mediate the horizontal transfer of resistance determinants. All PCRs were conducted using primers and cycling conditions detailed in Table S1. Methodology followed protocols previously established in the literature [33,34,37].

### 4.6. Virulence Genes Investigation

The presence of six key virulence genes in *Escherichia coli* isolates was investigated by polymerase chain reaction (PCR). These genes included: *fimH* (type 1 fimbrial adhesin), *ireA* (iron-responsive element), *fyuA* and *iutA* (iron acquisition systems), *hma* (heme acquisition protein), and *cnf1* (cytotoxic necrotizing factor 1). Primer sequences and annealing temperatures are listed in Table 1, and cycling conditions followed protocols previously described in the literature [31,38].

Genomic DNA was extracted from fresh bacterial growth on Mueller-Hinton agar plates. A full bacteriological loop of colonies was resuspended in sterile water and subjected to sonication for cell lysis using a two-step procedure: three cycles of 30 s of sonication followed by 10 s of vortexing and centrifugation at 13,000 rpm for 3 min. DNA-containing supernatants were transferred to sterile 5 mL polypropylene tubes (Eppendorf) and stored at −20 °C until use. This extraction protocol was based on the Standard Operating Procedure CCBH-t-034 from the Hospital Infection Research Laboratory (LAPIH/IOC/Fiocruz, Rio de Janeiro, Brazil).

PCRs were performed in 25 μL volumes containing 12.5 μL of MasterMix^®^ (Taq DNA polymerase, reaction buffer, dNTPs, MgCl_2_), 8.5 μL of ultrapure Milli-Q water, and 4 μL of extracted DNA template (adapted from Karam, 2018 [38]). Thermal cycling conditions were as follows: initial denaturation at 94 °C for 3 min; 30 cycles of denaturation at 94 °C for 1 min, annealing at gene-specific temperatures (see Table 1) for 1 min, and extension at 72 °C for 3 min; followed by a final extension at 72 °C for 3 min. Positive controls consisted of previously sequenced study isolates known to harbor the target genes: UPEC 364 (*fyuA*), UPEC 422 (*hma*), UPEC 366 (*iutA* and *fimH*), and UPEC 398 (*cnf1*). PCR amplicons were resolved by electrophoresis in 1.5% agarose gels (Invitrogen) prepared with 0.4X TBE buffer (containing 0.5 M EDTA pH 8.0, 1 M Tris pH 8.0, and 0.035 M boric acid), and run at 20 V/cm for 90 min. Gels were stained with GelRed^®^ (0.5 g/L), visualized under UV illumination, and imaged using an LPixEX transilluminator (Loccus Biotechnology, Morrisville, NC, USA). Selected amplicons were purified using the GE Healthcare Ilustra GFX PCR DNA purification kit (Chicago, IL, USA) and subjected to Sanger sequencing on an ABI 3730 DNA Analyzer (Applied Biosystems, Foster City, CA, USA). Sequence identity was confirmed by BLASTN (version 2.2.29) searches against the NCBI nucleotide database. Sequences were aligned to reference genes using the Geneious^®^ software (Version Prime 2025.1, “Map to Ref” tool), and homology was assessed via NCBI’s BLAST platform.

### 4.7. Sequence-Type and Phylogenetic Groups Determination

Total genomic DNA was extracted using a thermal lysis method with Chelex 100 resin (Bio-Rad, Hercules, CA, USA), as previously described [17]. To determine sequence types (STs), a multiplex PCR assay was employed to detect the most common UPEC clones: ST131, ST95, ST73, and ST69 [39]. In parallel, phylogenetic grouping was performed using a quadriplex PCR targeting the *chuA*, *yjaA*, *TspE4.C2*, and *arpA* genes, according to the revised Clermont method [40]. For sequence-type identification, PCRs were carried out in a final volume of 10 μL, containing 5 μL of MasterMix (Promega^®^, Madison, WI, USA), 0.2 μL of each primer (10 pmol/μL), 2.4 μL of DNase-free water (Thermo Fisher Scientific, Waltham, MA, USA), and 1 μL of DNA template per reaction. The cycling conditions were as follows: initial denaturation at 94 °C for 3 min; 30 cycles of amplification at 94 °C for 30 s, 60 °C for 30 s, and 72 °C for 30 s; followed by a final extension at 72 °C for 5 min. Primer sequences, target genes, and expected amplicon sizes are detailed in Table 2 (sequence typing) and Table 3 (phylogenetic grouping).

Phylogenetic grouping of *Escherichia coli* sensu stricto (A, B1, B2, C, D, E, F) was performed following bacterial culture on Trypticase Soy Agar (TSA) at 35 °C for 16–24 h. Approximately 3 isolated colonies were suspended in 100 µL of sterile Milli-Q water (Millipore, Burlington, MA, USA) and lysed by incubation at 100 °C for 10 min. PCRs were assembled in a total volume of 12 µL, containing 5.0 µL of MasterMix Green (Invitrogen, Carlsbad, CA, USA), 2.0 µL of lysate, 0.5 µL of each primer (20 pmol), and sterile water to volume. Thermal cycling conditions comprised an initial denaturation at 94 °C for 4 min, followed by 30 cycles of 94 °C for 5 s and annealing at 59 °C for 20 s, with a final extension at 72 °C for 5 min. Amplicons (2 µL) were resolved by electrophoresis on a 1.5% agarose gel stained with ethidium bromide (2 µL), run at 100 V for approximately 100 min. Phylogroup assignment was conducted according to the Clermont et al. (2013) [40] protocol.

### 4.8. Tenebrio Molitor Infection Model

The pathogenicity of the bacterial isolate UPEC 355 was evaluated using the Tenebrio molitor infection model [40]. UPEC 355 was selected as a representative virulent strain based on prior characterization, including strong biofilm formation and the presence of virulence genes *fyuA*, *iutA*, *fimH*, *cnf1*, and *hma* detected by PCR. Larvae were carefully selected based on size (~2.5 cm) and coloration, excluding brown-colored individuals. The bacterial inoculum ranged from 10^3^ to 10^8^ CFU. Larval mortality was assessed at these two inoculum concentrations. UPEC 355 was cultured in Luria–Bertani broth (LBB) at 37 °C for 24 h, yielding inoculum doses ranging from 2.6 × 10^3^ to 2.6 × 10^8^ CFU/mL. Each larva received 10 μL of the bacterial suspension by injection between the second and third body segments using a 0.25 mm microsyringe. Inoculated larvae were maintained in Petri dishes without food, protected from light, and incubated at 37 °C for up to 72 h. Melanized larvae were counted daily as an indicator of infection. Experiments were performed in triplicate, with 15 larvae per group. Larvae injected with sterile saline served as controls. Survival curves for each group were generated using the Kaplan–Meier method with GraphPad Prism software (v.7).

## 5. Conclusions

Overall, our findings demonstrate that UPEC isolates possess critical mechanisms for adhesion, iron acquisition, and host cell damage. Strains recovered from both inpatient and outpatient urine samples in Rio de Janeiro carried a diverse array of antimicrobial resistance genes alongside key virulence factors essential for the pathogenesis of urinary tract infections. Notably, the *in vivo* *Tenebrio molitor* infection model confirmed the pathogenic potential of an MDR UPEC strain, showing concentration-dependent lethality. These results support the notion that the combination of virulence and resistance traits can synergistically enhance the pathogenicity of specific UPEC clones. The detection of high-risk clones, particularly ST131 within phylogroup B2, raises significant concerns regarding their dissemination not only in healthcare settings but also in the broader community. These findings underscore the urgent need for strengthened surveillance and containment strategies to limit the spread of virulent and multidrug-resistant *E. coli* strains.

## Figures and Tables

**Table 1:** Sequence of primers used in the PCRs of the present study.

Primers Sequences		Annealing Temperature (°C)	Expected Fragment Size (bp)
(1) *irea*A			
F	5′-ATGAAGAACAAATATATC-3′	53 ^R1^	2500
R	5′-GAAGGATACTCTTACATT-3′		
(2) *fyu*A			
F	5′-ATGAAAATGACACGGCT-3′	56 ^R1^	2200
R	5′-GAAGAAATCAATTCGCG-3′		
(3) *hma*			
F	5′-ATGGTTAAAGATACAATC-3′	56 ^R1^	1850
R	5′-CCACTGATAACGGGTAT-3′		
(4) *iut*A			
F	5′-ATGAAAATGACACGGCT-3′	60 ^R1^	1750
R	5′-GAAGAAATCAATTCGCG-3′		
(5) *fim*H			
F	5′-TGCAGAACGGATAAGCCGTGG-3′	60 ^R2^	508
R	5′-GCAGTCACCTGCCCTCCGGTA-3′		
(6) *cnf*1			
F	5′-AAGATGGAGTTTCCTATGCAG-3′	54 ^R2^	543
R	5′-TCAGAGTCCTGCCCTCATTAT-3′		

R1: according to Karam, 2018 [38]; R2: according to Basu, 2013 [31]; °C (degrees Celsius); bp: base pairs.

**Table 2:** Multiplex PCR primers for Sequence types.

ST Target	Primer Name	Primers Sequences	Fragment Size (bp)
ST73	ST73_for	TGGTTTTACCATTTTGTCGGA	490
	ST73_rev	GGAAATCGTTGATGTTGGCT	
ST131	ST131_for	GACTGCATTTCGTCGCCATA	310
	ST131_rev	CCGGCGGCATCATAATGAAA	
ST95	ST95_for	ACTAATCAGGATGGCGAGAC	200
	ST95_rev	ATCACGCCCATTAATCCAGT	
ST69	ST69_for	ATCTGGAGGCAACAAGCATA	104
	ST69_rev	AGAGAAAGGGCGTTCAGAAT	

**Table 3:** Interpretation of PCR results for determining *E. coli sensu stricto* phylogroups.

Phylogroups of *E. coli sensu stricto*	Presence of PCR-Generated Fragments			
	*arpA*	*chuA*	*yjaA*	*TspE4.C2*
A	+	−	−	−
	+	−	+	−
B1	+	−	+	+
B2	−	+	+	+
	−	+	+	−
	−	+	−	+
C	+	−	+	−
D	+	+	−	−
	+	+	−	+
E	+	+	−	−
	+	+	−	+
	+	+	+	−
F	−	+	−	−

Legend: +: presence/positive PCR; −: absence/negative PCR.

## Data Availability

Data are contained within the article and Supplementary Materials.

## Supplementary Materials

The following supporting information can be downloaded at: https://www.mdpi.com/article/10.3390/antibiotics14090869/s1. Table S1: Primer sequences, PCR cycling conditions, and expected amplicon sizes used for the detection of antimicrobial resistance genes in *Escherichia coli* isolates; Table S2: Characteristics of the uropathogenic *Escherichia coli* (UPEC) isolates analyzed in this study.

## Abbreviations

The following abbreviations are used in this manuscript:

UTI	Urinary tract infection
UPEC	Uropathogenic *Escherichia coli*
STs	sequence types
PCR	polymerase chain reaction
CFU	colony-forming units
mL	milliliter
NG	Not-grouped
MDR	multidrug-resistant isolate
EMS	Emergency service
OS	Outpatient service
DC	dialysis center
MSC	male surgical clinic
NICU	neonatal intensive care unit
FSC	female surgical clinic
MixSC	mixed surgical clinic
h	hour
CNF1	cytotoxic necrotizing factor 1
MALDI-TOF MS	Matrix-Assisted Laser Desorption/Ionization Time-of-Flight mass spectrometry
μL	microliter
ODc	cutoff value
OD	optical density
SD	standard deviation
CLSI	Clinical & Laboratory Standards Institute
LBB	Luria–Bertani broth

## References

[1] Campos A.C.C., Andrade N.L., Ferdous M., Chlebowicz M.A., Santos C.C., Correal J.C.D., Lo Ten Foe J.R., Rosa A.C.P., Damasco P.V., Friedrich A.W. (2018). Comprehensive Molecular Characterization of *Escherichia coli* Isolates from Urine Samples of Hospitalized Patients in Rio de Janeiro, Brazil. Front. Microbiol..

[2] Pereira J.L., Volcão L.M., Klafke G.B., Vieira R.S., Gonçalves C.V., Ramis I.B., da-Silva P.E.A., von Groll A. (2019). Antimicrobial resistance and molecular characterization of extended-spectrum β-lactamases of *Escherichia coli* and *Klebsiella* spp. isolates from urinary tract infections in Southern Brasil. Microb. Drug Resist..

[3] Nasrollahian S., Halaji M., Hosseini A., Teimourian M., Armaki M.T., Rajabnia M., Gholinia H., Pournajaf A. (2022). Genetic Diversity, Carbapenem Resistance Genes, and Biofilm Formation in UPEC Isolated from Patients with Catheter-Associated Urinary Tract Infection in North of Iran. Int. J. Clin. Pract..

[4] Wagenlehner F.M.E., Bjerklund Johansen T.E., Cai T., Koves B., Kranz J., Pilatz A., Tandogdu Z. (2020). Epidemiology, definition and treatment of complicated urinary tract infections. Nat. Rev. Urol..

[5] Terlizzi M.E., Gribaudo G., Maffei M.E. (2017). Uropathogenic *Escherichia coli* (UPEC) Infections: Virulence Factors, Bladder Responses, Antibiotic, and Non-Antibiotic Antimicrobial Strategies. Front. Microbiol..

[6] Yang X., Chen H., Zheng Y., Qu S., Wang H., Yi F. (2022). Disease burden and long-term trends of urinary tract infections: A worldwide report. Front. Public Health.

[7] Reis A.C.C., Santos S.R.S., Souza S.C., Saldanha M.G., Pitanga T.N., Oliveira R.R. (2016). Ciprofloxacin resistance pattern among bacteria isolated from patients with community-acquired urinary tract infection. Rev. Inst. Med. Trop. Sao Paulo.

[8] Kumar S., Dave A., Wolf B., Lerma E.V. (2015). Urinary tract infections. Dis. Mon..

[9] Galindo-Méndez M. (2018). Molecular characterization and antimicrobial susceptibility pattern of extended-spectrum β-lactamase-producing *Escherichia coli* as cause of community acquired urinary tract infection. Rev. Chil. Infectol..

[10] Antimicrobial Resistance Collaborators (2022). Global burden of bacterial antimicrobial resistance in 2019: A systematic analysis. Lancet.

[11] Ho C.S., Wong C.T.H., Aung T.T., Lakshminarayanan R., Mehta J.S., Rauz S., McNally A., Kintses B., Peacock S.J., de la Fuente-Nunez C. (2025). Antimicrobial resistance: A concise update. Lancet Microbe.

[12] Gillings M.R. (2014). Integrons: Past, present, and future. Microbiol. Mol. Biol. Rev..

[13] Gündoğdu A., Long Y.B., Vollmerhausen T.L., Katouli M. (2011). Antimicrobial resistance and distribution of *sul* genes and integron-associated *int*I genes among uropathogenic *Escherichia coli* in Queensland, Australia. J. Med. Microbiol..

[14] Keshavarz M., Jo Y.H., Patnaik B.B., Park K.B., Ko H.J., Kim C.E., Edosa T.T., Lee Y.S., Han Y.S. (2020). TmRelish is required for regulating the antimicrobial responses to *Escherichia coli* and *Staphylococcus aureus* in *Tenebrio molitor*. Sci. Rep..

[15] Grape M., Sundström L., Kronvall G. (2003). Sulphonamide Resistance Gene *sul3* Found in *Escherichia coli* Isolates from Human Sources. J. Antimicrob. Chemother..

[16] Muhammed Ali R.A., Alshara J.M.R., Tuwaij S.S., Al-Khilkhali H.J.B. (2022). Study of Antibacterial Chemical Substances and Molecular Investigation among Sulfamethoxazole-Trimethoprim (SXT)-Resistant *Escherichia coli* Isolates. Rep. Biochem. Mol. Biol..

[17] Rahman M.M., Hossain M.M.K., Rubaya R., Halder J., Karim M.E., Bhuiya A.A., Khatun A., Alam J. (2022). Association of Antibiotic Resistance Traits in Uropathogenic *Escherichia coli* (UPEC) Isolates. Can. J. Infect. Dis. Med. Microbiol..

[18] Chin J.J., Lee H.M., Lee S.Y., Lee Y.Y., Chew C.H. (2024). High Carriage of *tetA*, *sul1*, *sul2* and *bla*_TEM_ Resistance Genes among the Multidrug-resistant Uropathogenic *Escherichia coli* (UPEC) Strains from Malaysian Patients. Trop. Life Sci. Res..

[19] Cardoso A.M., Flores V.R., do Rosario G.G., Succar J.B., Berbert L.C., Oliveira M.C.d.F., Canellas A.L.B., Laport M.S., Souza C.R.V.M., Chagas T.P.G. (2025). Antimicrobial Susceptibility of *Escherichia coli* Isolates Causing Community-Acquired Urinary Tract Infections: Comparison of Methods. Microorganisms.

[20] Soto S.M., Smithson A., Horcajada J.P., Martinez J.A., Mensa J.P., Vila J. (2006). Implication of biofilm formation in the persistence of urinary tract infection caused by uropathogenic *Escherichia coli*. Clin. Microbiol. Infect..

[21] Baldiris-Avila R., Montes-Robledo A., Buelvas-Montes Y. (2020). Phylogenetic Classification, Biofilm-Forming Capacity, Virulence Factors, and Antimicrobial Resistance in Uropathogenic *Escherichia coli* (UPEC). Curr. Microbiol..

[22] Yun K.W., Kim H.Y., Park H.K., Kim W., Lim I.S. (2014). Virulence factors of uropathogenic *Escherichia coli* of urinary tract infections and asymptomatic bacteriuria in children. J. Microbiol. Immunol. Infect..

[23] Flores C., Ling J., Loh A., Maset R.G., Aw A., White I.J., Fernando R., Rohn J.L. (2023). A human urothelial microtissue model reveals shared colonization and survival strategies between uropathogens and commensals. Sci. Adv..

[24] Lindblom A., Kiszakiewicz C., Kristiansson E., Yazdanshenas S., Kamenska N., Karami N., Åhrén C. (2022). The impact of the ST131 clone on recurrent ESBL-producing *E. coli* urinary tract infection: A prospective comparative study. Sci. Rep..

[25] Li D., Elankumaran P., Kudinha T., Kidsley A.K., Trott D.J., Jarocki V.M., Djordjevic S.P. (2023). Dominance of *Escherichia coli* sequence types ST73, ST95, ST127 and ST131 in Australian urine isolates: A genomic analysis of antimicrobial resistance and virulence linked to F plasmids. Microb. Genom..

[26] Hagelueken G., Duthie F.G., Florin N., Schubert E., Schiemann O. (2015). Expression, Purification and Spin Labelling of the Ferrous Iron Transporter FeoB from *Escherichia coli* BL21 for EPR Studies. Protein Expr. Purif..

[27] Gatya Al-Mayahie S.M., Al-Guranie D.R.T., Hussein A.A., Bachai Z.A. (2022). Prevalence of common carbapenemase genes and multidrug resistance among uropathogenic *Escherichia coli* phylogroup B2 isolates from outpatients in Wasit Province/Iraq. PLoS ONE.

[28] Talebi M., Najar-Peerayeh S., Bakhshi B. (2020). Hidden carbapenem resistance in the community-and hospital-associated OXA-48 gene-carrying uropathogenic *Escherichia coli*. Gene Rep..

[29] Lau S.H., Reddy S., Cheesbrough J., Bolton F.J., Willshaw G., Cheasty T., Fox A.J., Upton M. (2008). Major uropathogenic *Escherichia coli* strain isolated in the northwest of England identified by multilocus sequence typing. J. Clin. Microbiol..

[30] Johnson J.R., Clabots C., Porter S.B., Bender T., Johnston B.D., Thuras P. (2022). Intestinal Persistence of Colonizing *Escherichia coli* Strains, Especially ST131-H30, in Relation to Bacterial and Host Factors. J. Infect. Dis..

[31] Basu S., Mukherjee S.K., Hazra A., Mukherjee M. (2013). Molecular Characterization of Uropathogenic *Escherichia coli*: Nalidixic Acid and Ciprofloxacin Resistance, Virulent Factors and Phylogenetic Background. J. Clin. Diagn. Res..

[32] Alghoribi M.F., Gibreel T.M., Dodgson A.R., Beatson S.A., Upton M. (2014). *Galleria mellonella* infection model demonstrates high lethality of ST69 and ST127 uropathogenic *E. coli*. PLoS ONE.

[33] Canellas A.L.B., Lopes I.R., Mello M.P., Paranhos R., de Oliveira B.F.R., Laport M.S. (2021). *Vibrio* Species in an Urban Tropical Estuary: Antimicrobial Susceptibility, Interaction with Environmental Parameters, and Possible Public Health Outcomes. Microorganisms.

[34] Alves G.D.S.O., Canellas A.L.B., Gallo M.N., Vinzon S.B., Laport M.S. (2024). In treacherous waters: Detection of colistin-resistant bacteria in water and plastic litter from a recreational estuary. Lett. Appl. Microbiol..

[35] Stepanović S., Vuković D., Hola V., Di Bonaventura G., Djukić S., Cirković I., Ruzicka F. (2007). Quantification of biofilm in microtiter plates: Overview of testing conditions and practical recommendations for assessment of biofilm production by staphylococci. APMIS.

[36] CLSI (Clinical and Laboratory Standards Institute) (2020). Performance Standards for Antimicrobial Susceptibility Testing. CLSI Supplement M100.

[37] Richter L., Plessis E.M., Duvenage S., Korsten L. (2020). Occurrence, Phenotypic and Molecular Characterization of Extended-Spectrum- and AmpC- β-Lactamase Producing Enterobacteriaceae Isolated from Selected Commercial Spinach Supply Chains in South Africa. Front. Microbiol..

[38] Karam M.R.A., Habibi M., Bouzari S. (2018). Relationships between virulence factors and antimicrobial resistance among *Escherichia coli* isolated from urinary tract infections and commensal isolates in Tehran, Iran. Osong Public Health Res. Perspect..

[39] Doumith M., Day M., Ciesielczuk H., Hope R., Underwood A., Reynolds R., Wain J., Livermore D.M., Woodford N. (2015). Rapid identification of major *Escherichia coli* sequence types causing urinary tract and bloodstream infections. J. Clin. Microbiol..

[40] Clermont O., Christenson J.K., Denamur E., Gordon D.M. (2013). The Clermont *Escherichia coli* phylo-typing method revisited: Improvement of specificity and detection of new phylo-groups. Environ. Microbiol. Rep..

