# Supplementary material for: Assessment of Antimicrobial Resistance and Virulence of Biofilm-Forming Uropathogenic Escherichia coli from Rio de Janeiro
Source: Antibiotics (Basel). 2025 Aug 29;14(9):869. doi: 10.3390/antibiotics14090869 (PMC12466447; doi:10.3390/antibiotics14090869)
Supplement: Supplementary file 1 [file antibiotics-14-00869-s001.zip › antibiotics-3720534-supplementary.docx]

**Table S1.** Primers, cycling conditions and amplicon sizes used in the PCR reactions for antimicrobial resistance genes.

| Resistance | Gene | | Primers sequences | | | Cycle conditions | | | | Amplicon sizes  in base pairs | |  |
| --- | --- | --- | --- | --- | --- | --- | --- | --- | --- | --- | --- | --- |
|  | *bla_CTX-M-1,2_* | | 5’-ATGTGCAGYACCAGTAA-3’  5’-CGCTGCCGGTTTTATCSCCC-3’ | | | Denaturation 95ºC/10 min  30 cycles of 95ºC/30 s, 57ºC/30 s, 72ºC/45 s  Final extension 72ºC/10 min | | | | 512 | |  |
| *β-lactams* | *bla_CTX-M-8_* | | 5’-AACRCRCAGACGCTCTAC-3’  5’-TCGAGCCGGAASGTGTYAT-3’ | | |  |  |  |  | 333 | |  |
|  | *bla_CTX-M-14_* | | 5’-GGTGACAAAGAGARTGCAACGGAT-3'  5’-TTACAGCCCTTCGGCGATGA-3' | | |  |  |  |  | 876 | |  |
|  | *bla_TEM_* | | 5’-CCCTTATTCCCTTTYTTGCGG-3’  5’-AACCAGCCAGCCWGAAGG-3’ | | |  |  |  |  | 650 | |  |
|  | *bla_GES_* | | 5’-AGCAGCTCAGATCGGTGTTG-3’  5’-CCGTGCTCAGGATGAGTTG-3’ | | |  |  |  |  | 750 | |  |
|  | *bla_SHV_* | | 5’-CTTGACCGCTGGGAAACGG-3’  5’-AGCACGGAGCGGATCAACGG-3’ | | |  |  |  |  | 200 | |  |
|  | *bla*_KPC_ | | 5’-GCTACACCTAGCTCCACCTTC-3’  5’- TGGAGGGCCAATAGATGATT-3’ | | | Denaturation 94ºC/5 min  30 cycles of 94ºC/1 min, 61ºC/30 s, 72ºC/1 min  Final extension 72ºC/5 min | | | | 950 | |  |
| *Quinolones* | *qnrA* | | 5’-AGAGGATTTCTCACGCCAGG-3’  5’-TGCCAGGCACAGATCTTGAC-3’ | | |  | | | | 580 | |  |
|  | *qnrD* | | 5'-ATGGAAAAGCACTTTATCAATGAAAAG-3'  5'-TTATCGGTGAACAATAACACCTAAAC-3' | | | Denaturation 95ºC/10 min  25 cycles of 95ºC/45 s, 58ºC/45 s,72ºC/15 s  Final extension 72ºC/3 min | | | | 691 | |  |
|  | *qnrS* | | 5'-ACGACATTCGTCAACTGCAA-3'  5’-TAAATTGGCACCCTGTAGGC-3' | | |  | | | | 428 | |  |
| *Aminoglycosides* | *aac(6′)-Ie-aph(2′)-Ia* | | 5’-GCCAGAACATGAATTACACGAG-3’  5’-CTGTTGTTGCATTTAGTCTTTCC-3’ | | | Denaturation 95ºC/2 min  30 cycles of 95ºC/30 s; 53,5ºC/30 s; 72ºC/30 s  Final extension 72ºC/5 min | | | | 611 | |  |
|  | *aac(6′)-Ib* | | 5’-TTGCGATGCTCTATGAGTGGCTA-3’  5’-CTCGAATGCCTGGCGTGTTT-3’ | | | Denaturation 95ºC/10 min  25 cycles of 95ºC/45 s, 58ºC/45 s,72ºC/15 s  Final extension 72ºC/3 min | | | | 482 | |  |
|  | *ant(2”)-I* | | 5’-TGGGCGATCGATGCACGGCTRG-3’  5’-AAAGCGGCACGCAAGACCTCMAC-3’ | | | Denaturation 94ºC/10 min  35 cycles of 94ºC/1 min, 58ºC/1 min, 72ºC/1 min  Final extension 72ºC/10 min | | | | 428 | |  |
| *Integrase-integrons* | *intl1* | | 5’-GGCTTCGTGATGCCTGCTT-3’  5’-CATTCCTGGCCGTGGTTCT-3’ | | | Denaturation 94ºC/10 min  35 cycles of 98ºC/30 s, 60ºC/30 s, 72ºC/30 s  Final extension 72ºC/10 min | | | | 146 | |  |
|  | *intl2* | | 5’-CACGGATATGCGACAAAAAGG-3’  5’-TGTAGCAAACGAGTGACGAAATG-3’ | | | Denaturation 94ºC/12 min  30 cycles of 94ºC/30 s; 60ºC/30 s; 72ºC/1 min  Final extension 72ºC/8 min | | | | 788 | |  |
|  | *intl3* | | 5’-AGTGGGTGGCGAATGAGTG-3’  5’-TGTTCTTGTATCGGCAGGTG-3’ | | | Denaturation 94ºC/12 min  30 cycles of 94ºC/30 s; 60ºC/30 s; 72ºC/1 min  Final extension 72ºC/8 min | | | | 600 | |  |
| *Sulfonamides* | *sul1*  *sul2* | | 5’- CGGCGTGGGCTACCTGAACG -3’  5’- GCCGATCGCGTGAAGTTCCG -3’  5’- GCGCTCAAGGCAGATGGCATT -3’  5’- GCGTTTGATACCGGCACCCGT -3’ | | | Denaturation 94°C/ 5 min  30 cycles of 94°C/ 15s, 69°C/ 30s, 72°C/ 60s  Final extension at 72°C/ 7 min | | | | 433  293 | |  |
|  | *sul3* | | 5’- GAGCAAGATTTTTGGAATCG -3’  5’- CTAACCTAGGGCTTTGGATAT -3’ | | | Denaturation 94°C/ 5 min  35 cycles of 94ºC/40s, 53ºC/40s, 72ºC/30s  Final extension at 72ºC/ 1 min | | | | 750 | |  |
|  |  | |  | | |  | | | |  | |  |
|  |  | | | |  | | |  |  |  |  |  |
|  |  | | | |  | | |  |  |  |  |  |
|  | | *mcr-1* | | 5′-ATGCCAGTTTCTTTCGCGTG-3′  5′-TCGGCAAATTGCGCTTTTGGC-3′ | | |  | | | | 502 | |
|  | | *mcr-2* | | 5′-GATGGCGGTCTATCCTGTAT-3′  5′- AAGGCTGACACCCCATGTCAT-3’ | | |  | | | | 379 | |
| *Colistin* | | *mcr-3* | | 5′-ACCAGTAAATCTGGTGGCGT-3′  5′- AGGACAACCTCGTCATAGCA-3′ | | | Denaturation 94°C/ 4 min  30 cycles of 94°C/ 5s, 59°C/ 20s, 72°C/ 30s  Final extension at 72°C/ 5 min | | | | 296 | |
|  | | *mcr-4* | | 5′-TTGCAGACGCCCATGGAATA-3′  5′-GCCGCATGAGCTAGTATCGT-3′ | | |  | | | | 207 | |
|  | | *mcr-5* | | 5′- GGACGCGACTCCCTAACTTC-3′  5′-ACAACCAGTACG AGAGCACG-3′ | | |  | | | | 608 | |

**Table S2.** Characteristics of 50 Uropathogenic *Escherichia coli* strains of the study.

| Strain code | Isolation  date | Hospital Sector | | Gender | | Age | Virulence | | Antimicrobial resistance | | |  | |  |
| --- | --- | --- | --- | --- | --- | --- | --- | --- | --- | --- | --- | --- | --- | --- |
|  |  |  |  | |  | | **Virulence gene pattern** | **Biofilm-production level** | **Resistance gene pattern** | | **Disk diffusion CLSI/MDR** | | **Phylogroup** | |
| 353 | 05/20//2019 | SEM | F | | 15 | | *fyuA, iutA, fimH, hma* | Moderate | *tem* | | NAL AMP CIP GEN LVX NOR MDR | | B2 | |
| 354 | 05/20//2019 | SAM | | F | | 23 | *fyuA, iutA, fimH, cnf1* | Moderate | *int1 tem sul2* | AMP SUT TET  MDR | | D/E | |  |
| 355 | 05/21//2019 | SAM | | F | | 79 | *fyuA, iutA, fimH, cnf1,hma* | Moderate | *ctx-m 1,2 tem* | NAL AMP APS ATM CFZ COM CTX CRO CRX CIP LVX NOR  MDR | | D/E | |  |
| 356 | 05/21//2019 | SAM | | F | | 61 | *fyuA, iutA, fimH, cnf1* | Moderate | *sul1* | AMP ATM SUT TET  MDR | | F | |  |
| 358 | 05/22//2019 | SAM | | F | | 75 | *fyuA, iutA, fimH, hma,* | Strong | *int1 tem sul1 sul2* | NAL AMP CIP GEN LVX NOR SUT TET TOB  MDR | | B2 | |  |
| 359 | 06/04/2019 | SEM | | F | | 26 | *fyuA, iutA, fimH, cnf1* | Moderate | *tem qnrs sul1 sul2* | NAL AMP SUT TET  MDR | | D/E | |  |
| 363 | 06/04/2019 |  | | F | | 41 | *fyuA, fimH, cnf1* | Strong | *sul1* | NAL AMI AMP CIP GEN LVX NOR SUT TET  MDR | | D/E | |  |
| 364 | 06/04/2019 | SAM | | F | | 92 | *fyuA, iutA, fimH, cnf1, hma,* | Moderate | *sul1* | Susceptible | | D/E | |  |
| 365 | 06/04/2019 | SAM | | F | | 76 | *iutA, cnf1* | Moderate | *ges sul1* | NAL FOS NIT TET TOB  MDR | | A | |  |
| 366 | 06/04/2019 | SAM | | F | | 53 | *fyuA, fimH, cnf1, hma,* | Strong | *int1 sul1* | NAL NOR TET  MDR | | B2 | |  |
| 367 | 06/04/2019 | SAM | | F | | 62 | *iutA, fimH,* | Moderate | *tem sul2* | NAL AMP FOS NIT TET  MDR | | D/E | |  |
| 368 | 06/04/2019 | SAM | | F | | 67 | *fyuA, fimH, hma* | Strong | *sul1 sul2* | NAL CIP FOS LVX NIT SUT  MDR | | D/E | |  |
| 397 | 06/10/2019 | SAM | | M | | 69 | *fyuA, fimH, cnf1, hma,* | Strong | *sul1* | LVX | | B2 | |  |
| 398 | 06/10/2019 | SAM | | M | | 73 | *fyuA, fimH, cnf1, hma,* | Strong | *sul1* | NAL TOB | | B2 | |  |
| 399 | 06/10/2019 | SAM | | M | | 87 | *fyuA, fimH, hma,* | Moderate | *int1 tem sul1 sul2* | NAL AMP CFZ CIP GEN LVX NOR SUT TOB  MDR | | D/E | |  |
| 421 | 06/17/2019 | SAM | | F | | 81 | *cnf1* | Weak | *int1 tem qnrs sul1* | AMP TET | | A | |  |
| 422 | 06/17/2019 | CD | | M | | 57 | *fyuA, fimH, cnf1, hma,* | Strong | *tem sul1* | AMP TET | | B2 | |  |
| 423 | 06/17/2019 | SEM | | F | | 70 | *fyuA, fimH, cnf1* | Weak | *sul2* | Susceptible | | D/E | |  |
| 424 | 06/17/2019 | SEM | | F | | 33 | *fimH, cnf1* | Strong | *none* | NAL AMP APS CFZ CIP GEN LVX NOR SUT TET TOB MDR | | A | |  |
| 425 | 06/17/2019 | SAM | | F | | 38 | *fyuA, iutA, fimH, cnf1* | Moderate | *int2 tem sul2* | NAL AMP APS CFZ SUT  MDR | | B2 | |  |
| 1 | 08/14/2019 | CD | | F | | 34 | *iutA, fimH* | Strong | *int1 ctx-m1,2 tem aac(6’)lb sul2* | AMC AMP APS ATM CFZ CTX CRO CRX CIP GEN SUT TET TOB MDR | | A | |  |
| 2 | 08/14/2019 | SAM | | F | | 59 | *fyuA, iutA, fimH* | Moderate | *int1 tem sul1* | NAL AMP APS ATM CFZ CTX CFO CRO CRX CIP GEN LVX NOR SUT TOB  MDR | | B2 | |  |
| 3 | 08/09/2019 | SEM | | M | | 76 | *fyuA, iutA, fimH* | Strong | *tem sul2* | NAL AMP APS ATM CFZ CTX CRO CRX CIP GEN LVX NOR SUT TET TOB  MDR | | B2 | |  |
| 4 | 09/08/2019 | CMM | | M | | 65 | *fyuA, iutA, fimH* | Strong | *int1 tem sul1* | NAL AMP CFZ CIP LVX NOR SUT TET  MDR | | B2 | |  |
| 5 | 09/05/2019 | SEM | | F | | 62 | *fyuA, iutA, fimH* | Moderate | *tem sul2* | NAL AMP APS CFZ CTX CIP LVX NOR SUT TET  MDR | | B2 | |  |
| 6 | 08/15/2019 | UTINEO | | M | | 0 | *iutA, fimH* | Weak | *int1 ctx-m1,2 tem shv aac(6’) lb sul1* | NAL AMC AMP APS ATM CFZ COM CTX CRO CRX CIP LVX NOR SUT TET TOB  MDR | | D/E | |  |
| 7 | 08/15/2019 | SEM | | F | | 18 | *fyuA, fimH* | Moderate | *int2 ctx-m 1,2* | AMP APS CFZ COM CTX CRO CRX SUT TET  MDR | | B2 | |  |
| 8 | 10/16/2019 | SEM | | F | | 53 | *fyuA, iutA, fimH* | Moderate | *ctx-m 1,2 tem sul2* | AMP APS CFZ COM CTX CRO CRX SUT TET  MDR | | D/E | |  |
| 9 | 08/14/2019 | SEM | | F | | 58 | *fyuA, iutA, fimH* | Moderate | *int1 ctx-m 1,2 tem shv sul1 sul2* | NAL AMP ATM CFZ CTX CRO CRX CIP GEN LVX NOR SUT TET TOB  MDR | | B2 | |  |
| 10 | 08/26/2019 | SAM | | F | | 82 | *fyuA, iutA, fimH* | Moderate | *int1 sul1* | NAL AMP APS CFZ COM CTX CRO CRX CIP LVX NOR SUT TET MDR | | A | |  |
| 11 | 08/28/2019 | CD | | F | | 53 | *fyuA, iutA, fimH* | Moderate | *ctx-m 1,2 tem shv sul1* | NAL AMC AMP APS ATM CFZ COM CTX CRO CRX CIP FOS LVX NIT NOR SUT TET  MDR | | F | |  |
| 12 | 08/28/2019 | SAM | | F | | 67 | *fyuA, iutA, fimH* | Strong | *sul1* | NAL FOS NIT  MDR | | B2 | |  |
| 13 | 08/27/2019 | SEM | | F | | 63 | *fyuA, iutA, fimH, cnf1* | Strong | *sul1* | NAL FOS NIT  MDR | | F | |  |
| 15 | 08/20/2019 | SAM | | M | | 30 | *fyuA, iutA, fimH* | Strong | *tem sul2* | AMP CFZ TET | | B2 | |  |
| 16 | 09/10/2019 | CMF | | F | | 64 | *fyuA, iutA, fimH, cnf1* | Moderate | *tem shv sul2* | AMP APS CFZ | | B2 | |  |
| 17 | 09/10/2019 | CMF | | F | | 62 | *fyuA, fimH* | Weak | *ctx-m 1,2 aac(6’) lb sul1 sul2* | NAL AMI AMC AMP APS ATM CFZ COM CTX CRO CRX CIP LVX NOR PIT SUT TET TOB  MDR | | B2 | |  |
| 18 | 09/04/2019 | CMF | | F | | 39 | *fyuA, iutA, fimH* | Strong | *tem* | AMP | | D/E | |  |
| 19 | 09/04/2019 | CMF | | F | | 71 | *fyuA, iutA, fimH* | Weak | *sul1* | Susceptible | | B2 | |  |
| 20 | 09/04/2019 | CMF | | F | | 35 | *iutA, fimH* | Moderate | *none* | Susceptible | | D/E | |  |
| 21 | 09/04/2019 | CMF | | F | | 83 | *iutA, fimH* | Moderate | *int1 ctx-m1,2 tem aac(6‘)lb sul2* | NAL AMP APS ATM CFZ CTX CRO CRX CIP GEN SUT TET TOB MDR | | A | |  |
| 22 | 09/04/2019 | CMF | | F | | 34 | *fyuA, iutA, fimH, cnf1* | Moderate | *tem sul1* | AMP APS CFZ | | B2 | |  |
| 23 | 09/04/2019 | CMF | | F | | 67 | *fyuA, iutA, fimH, cnf1* | Moderate | *sul1* | Susceptible | | B2 | |  |
| 24 | 09/27/2019 | SEM | | F | | 64 | *fyuA, iutA, fimH, cnf1* | Moderate | *int1 tem sul1* | AMP TET | | B2 | |  |
| 25 | 09/26/2019 | CD | | F | | 2 | *fyuA, iutA, fimH, cnf1* | Weak | *int1 ctx-m1,2 aac(6’)lb sul1 sul2* | AMP APS CFZ | | B2 | |  |
| 26 | 10/02/2019 | SAM | | F | | 39 | *fyuA, fimH* | Weak | *int1 ctx-m1,2 shv* | NAL AMI AMC AMP APS ATM CFZ COM CTX CRO CRX CIP LVX NOR PIT SUT TET TOB  MDR | | A | |  |
| 27 | 09/26/2019 | SAM | | F | | 76 | *fyuA, iutA, fimH, cnf1* | Moderate | *int2 ctx-m1,2 tem shv aac(6’)lb sul1* | AMP | | D/E | |  |
| 28 | 10/09/2019 | CCEMI | | M | | 34 | *fyuA, iutA, fimH, cnf1* | Moderate | *tem sul1 sul2* | Susceptible | | D/E | |  |
| 31 | 10/30/2019 | SAM | | M | | 74 | *fyuA, fimH, cnf1* | Weak | *int1 tem* | AMP APS CFZ | | B2 | |  |
| 32 | 09/25/2019 | SAM | | F | | 10 | *fyuA, iutA, fimH* | Weak | *int1 tem sul2* | Susceptible | | B2 | |  |
| 33 | 10/08/2019 | SAM | | F | | 65 | *fyuA, iutA, fimH, cnf1* | Moderate | *none* | AMP TET | | B2 | |  |
